# Robust dose‐painting‐by‐numbers vs. nonselective dose escalation for non‐small cell lung cancer patients

**DOI:** 10.1002/mp.14840

**Published:** 2021-05-14

**Authors:** Steven F. Petit, Sebastiaan Breedveld, Jan Unkelbach, Dick den Hertog, Marleen Balvert

**Affiliations:** ^1^ Department of Radiotherapy Erasmus MC Cancer Institute University Medical Center Rotterdam Rotterdam The Netherlands; ^2^ Department of Radiation Oncology University Hospital Zürich Zürich Switzerland; ^3^ Department of Econometrics and Operations Research Tilburg University Tilburg The Netherlands

**Keywords:** nonselective dose escalation (NSDE), radiotherapy, robust dose‐painting‐by‐numbers (DPBN), treatment planning, tumor control probability (TCP), uncertainty‐based planning

## Abstract

**Purpose:**

Theoretical studies have shown that dose‐painting‐by‐numbers (DPBN) could lead to large gains in tumor control probability (TCP) compared to conventional dose distributions. However, these gains may vary considerably among patients due to (a) variations in the overall radiosensitivity of the tumor, (b) variations in the 3D distribution of intra‐tumor radiosensitivity within the tumor in combination with patient anatomy, (c) uncertainties of the 3D radiosensitivity maps, (d) geometrical uncertainties, and (e) temporal changes in radiosensitivity. The goal of this study was to investigate how much of the theoretical gains of DPBN remain when accounting for these factors. DPBN was compared to both a homogeneous reference dose distribution and to nonselective dose escalation (NSDE), that uses the same dose constraints as DPBN, but does not require 3D radiosensitivity maps.

**Methods:**

A fully automated DPBN treatment planning strategy was developed and implemented in our in‐house developed treatment planning system (TPS) that is robust to uncertainties in radiosensitivity and patient positioning. The method optimized the expected TCP based on 3D maps of intra‐tumor radiosensitivity, while accounting for normal tissue constraints, uncertainties in radiosensitivity, and setup uncertainties. Based on FDG‐PETCT scans of 12 non‐small cell lung cancer (NSCLC) patients, data of 324 virtual patients were created synthetically with large variations in the aforementioned parameters. DPBN was compared to both a uniform dose distribution of 60 Gy, and NSDE. In total, 360 DPBN and 24 NSDE treatment plans were optimized.

**Results:**

The average gain in TCP over all patients and radiosensitivity maps of DPBN was 0.54 ± 0.20 (range 0–0.97) compared to the 60 Gy uniform reference dose distribution, but only 0.03 ± 0.03 (range 0–0.22) compared to NSDE. The gains varied per patient depending on the radiosensitivity of the entire tumor and the 3D radiosensitivity maps. Uncertainty in radiosensitivity led to a considerable loss in TCP gain, which could be recovered almost completely by accounting for the uncertainty directly in the optimization.

**Conclusions:**

Our results suggest that the gains of DPBN can be considerable compared to a 60 Gy uniform reference dose distribution, but small compared to NSDE for most patients. Using the robust DPBN treatment planning system developed in this work, the optimal DPBN treatment plan could be derived for any patient for whom 3D intra‐tumor radiosensitivity maps are known, and can be used to select patients that might benefit from DPBN. NSDE could be an effective strategy to increase TCP without requiring biological information of the tumor.

## Graphical abstract

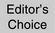

## 1 Introduction

Dose‐painting‐by‐numbers (DPBN) could potentially lead to a more effective radiation treatment than conventional treatment plans (see^1^ and references therein). Theoretical studies have shown huge gains for DPBN^2^, ^3^ and the first clinical trials are ongoing or have been completed (e.g., clinicaltrials.gov NCT01168479, NCT01341535, and NCT01024829).^4^

By far the largest challenge of DPBN is to measure spatial differences in radiosensitivity or likelihood of recurrence within the tumor. Imaging techniques and (combinations of) tracers that have been proposed to identify boost regions include PET with a variety of tracers such as ^18^F‐fluorodeoxyglucose (FDG), ^18^F‐fluoromisonidazole (FMISO), ^18^F‐fluorothymidine (FLT), ^18^F‐fluoroazomycin‐arabinofuranoside (FAZA), ^18^F‐flortanidazole (HX4), and ^68^Ga‐Glu‐urea‐Lys(Ahx)‐HBED‐CC (^68^Ga‐HBED‐CC PSMA) and various MRI sequences, such as diffusion‐weighted imaging (DWI), dynamic contrast‐enhanced (DCE) MRI, blood‐oxygen‐level‐dependent (BOLD) MRI, or MR spectroscopic imaging.^5^, ^6^, ^7^, ^8^, ^9^, ^10^, ^11^, ^12^, ^13^, ^14^

Two categories of methods can be distinguished to use these images to steer the dose painting treatment planning. The first method assigns a priori desired dose levels to different regions or voxels of the tumor based on imaging data. The treatment planning strategy then aims to minimize the difference between the desired and planned dose distribution for each voxel. A pragmatic approach is to define a boost region based on tracer uptake, assign a desired boost dose level, and optimize the treatment plan using a conventional simultaneous integrated boost technique.^15^ Others converted 3D tracer maps to assign a prescription dose or escalation factor to each voxel individually.^16^, ^17^, ^18^

The second method, which is the topic of the current study, aims to directly optimize the probability of tumor control. This requires conversion of 3D tracer maps to 3D maps of intra‐tumor radiosensitivity.^19^, ^20^, ^21^, ^22^, ^23^, ^24^ Theoretical studies that assumed that any dose level could be delivered to any voxel have shown that, if the radiosensitivity of each voxel is known, DPBN could lead to gains in tumor control probability (TCP) of more than 30 percentage points without increasing normal tissue exposure.^2^, ^3^

These gains are very promising, but the gains should be interpreted with caution since they may be affected by multiple patient‐specific factors.

1. The radiosensitivity of the tumor overall determines the difficulty of controlling the tumor regardless of treatment planning strategy: highly radiosensitive tumors can be controlled with a conventional dose distribution and therefore would not benefit from DPBN, while extremely radio‐resistant tumors could neither be controlled with a conventional dose distribution, nor with DPBN and therefore would not benefit either.
2. The 3D distribution of intra‐tumor radiosensitivity, in combination with the patient anatomy, should allow for a dose modulation that matches the variation in radiosensitivity within the tumor. DPBN can only be effective if the dose can be modulated according to the spatial differences in radiosensitivity, which depends on the spatial differences themselves and on the proximity of organs at risk. For instance, if the spatial differences are present primarily at length scales that are too small to modulate the radiation dose accordingly or if organs at risk (OARs) limit a high dose to resistant regions, DPBN would not be effective.
3. Since it is a huge challenge to derive 3D maps of intra‐tumor radiosensitivity, any estimates of these maps will be, to some extent, uncertain. Ignoring this uncertainty may lead to suboptimal dose distributions and could reduce the gains of DPBN.
4. Geometrical uncertainties will hamper precise delivery of dose according to spatial differences in radiosensitivity.
5. Temporal changes in the radiosensitivity across the tumor throughout the course of therapy could influence the potential benefit of DPBN as well.

Moreover, compared to conventional treatment planning, the benefit of dose painting stems from two factors. First, more dose to resistant and less to sensitive regions leads to a more effective use of dose. Second, to allow variations in dose within the tumor, DPBN uses higher maximum dose constraints to the tumor, compared to conventional treatment planning. A side effect of the higher maximum tumor dose is that gradients at the edge of the target can be steeper. The steeper gradients allow a higher integral tumor dose compared to conventional treatment planning for the same normal tissue constraints, and therefore a higher TCP. The TCP benefit that is attributed to this second factor could be achieved also without DPBN, simply by allowing a higher maximum tumor dose. So to determine the real added value of DPBN, only the benefit that stems from the first factor should be considered.

Therefore, we compared DPBN to a technique we refer to as nonselective dose escalation (NSDE). NSDE uses the same normal tissue, maximum tumor dose constraints, and optimization technique as DPBN, but assumes that all tumors and tumor subregions are equally radiosensitive. NSDE therefore does not require any patient/tumor/subregion‐specific information on radiosensitivity. Therefore, the difference between DPBN and NSDE solely stems from matching the dose to spatial differences in radiosensitivity. The exact implementation of NSDE is described in section 2.C.2.

The goal of this study was to investigate what remains of the theoretical gains of DPBN compared to NSDE, when accounting for the factors (i) through (v). For an unbiased comparison between NSDE and DPBN, a treatment planning strategy was required that (a) could directly optimize the TCP for both DPBN and NSDE, (b) was fully automated to avoid the inevitable bias of manual treatment planning, and (c) accounted for uncertainties in radiosensitivity distributions and patient positioning directly in the optimization. Such a strategy was developed and implemented in our treatment planning system (TPS) Erasmus‐iCycle.^25^ It was applied to DPBN and NSDE for a large range of patient anatomies, 3D radiosensitivity maps, uncertainty scenarios, and temporal changes in radiosensitivity.

## 2 MATERIALS AND METHODS

Section 2.A describes the development and implementation of the robust DPBN treatment plan optimization strategy. Section 2.B presents the patient data and experiments that were performed to investigate how DPBN depends on the factors (i) to (v) mentioned above. Section 2.C describes the treatment planning constraints for DPBN and NSDE, and Section 2.D gives an overview of the different treatment plans.

### 2.A Robust DPBN optimization based on TCP

For the DPBN optimization, we used a common formulation of TCP^26^:

(1) $$\begin{array}{cc} TCP\left(\alpha ,\rho ,D^{T}x\right) & =\prod_{i=1}^{N}VCP\left(\alpha_{i},\rho_{i},d_{i}^{T}x\right)\\ & =\prod_{i=1}^{N}\exp\left(‐\rho_{i}\;\exp\left(‐\alpha_{i}d_{i}^{T}x\right)\right), \end{array}$$

where ***α*** = (*α*_1_,…,*α*_N_) and ***ρ* **= (*ρ_1_*,…,*ρ_N_*) denote the 3D maps of intra‐tumor radiosensitivity. *VCP*(*α_i_,ρ_i_*,$d_{i}^{T}$,***x***) stands for the voxel control probability of voxel i, and hence reflects the voxel’s dose–response relation. The parameter ***α*** primarily affects the slope of the VCP curve while a change in ***ρ*** leads to a shift of the VCP curve. D denotes the dose‐influence matrix with columns ***d_i_***, and ***x*** denote the bixel intensities. Note that a beam can be virtually divided into beamlet elements of bixels by a grid, and the intensity of each bixel can be controlled. *N* is the number of voxels in the tumor. Throughout this paper, vectors are indicated in boldface, and matrices are denoted in upper case. The radiosensitivity parameters $\alpha_{i}$ and $\rho_{i}$ can be specified per voxel or per tumor region.

#### 2.A.1 Accounting for uncertainty in radiosensitivity

As current techniques do not allow for an exact determination of $\alpha_{i}$ and $\rho_{i}$, estimating them inevitably results in uncertainty on their values. We therefore accounted for such uncertainties directly into treatment plan optimization. For this purpose, the *expected* value of the TCP was optimized over probability distributions for ***α*** and ***ρ***. To yield a convex optimization problem, the log of the expectation was maximized instead of maximizing the expected TCP^27^, ^28^:

(2) $$\max_{x\geq 0}\text{log}\left(E_{\left(\alpha ,\rho \right)\in U}\left(TCP\left(\alpha ,\rho ,D^{T}x\right)\right)\right),$$

where U denotes the support of the density function (i.e., all values of ***α*** and ***ρ*** with nonzero probability). Note that taking the log does not alter the optimal solution.^27^, ^28^

For a continuous log‐concave density function on (***α***,***ρ***), optimization problem (2) is convex in ***x*** since the expectation of the TCP is log‐concave in ***x***.^29^ Deriving an explicit formulation of the objective function of (2) using a continuous density function for (***α***,***ρ***) is however not possible. Therefore, the probability density function was discretized to obtain a discrete support set U, so we use the following definition of the expected TCP:

(3) $$E_{\left(\alpha ,\rho \right)\in U}\left(TCP\left(\alpha ,\rho ,D^{T}x\right)\right)\approx \sum_{\left(\alpha ,\rho \right)\in U}p\left(\alpha ,\rho \right)TCP\left(\alpha ,\rho ,D^{T}x\right),$$

where $p\left(\cdot \right)$ denotes the density function of (***α***,***ρ***). We were unable to prove that optimization problem (2) is convex for a discrete density function on (***α***,***ρ***). However, for a sufficiently dense sample of values for ***α*** and ***ρ***, the density function gets close to continuous and problem (2) is expected to become (nearly) convex. Additionally the Erasmus‐iCycle solver is able to handle minor convexity violations.^30^ Details on the used 3D intra‐tumor radiosensitivity maps ***α*** and ***ρ*** and their probability density functions can be found in sections 2.B.3 and 2.B.4.

#### 2.A.2 Accounting for geometrical uncertainties

In conventional radiation therapy planning, positional uncertainties are accounted for using a planning target volume (PTV) margin. For dose painting, the required size of the margin between different regions would depend on the dose difference between the regions. However, this dose difference is not known a priori for DPBN based on TCP optimization. Therefore, in this study, the positional uncertainties were incorporated directly into the optimization.

Patient positional uncertainties were split into random and systematic positioning errors. Both were assumed to follow Gaussian distributions.^30^, ^31^ The random error was accounted for by convolving the pencil beam kernels with the Gaussian distribution of the random errors.^32^, ^33^, ^34^ To account for systematic errors, we included S scenarios in the optimization, each representing a rigid shift of the patient relative to the isocenter. The minimum expected TCP over these scenarios was then maximized:

(4) $$\underset{x\geq 0}{\text{max}}\underset{s\in \left\{0,\ldots ,S\right\}}{\text{min}}\text{log}\left(E_{\left(\alpha ,\rho \right)\in U}\left(TCP\left(\alpha ,\rho ,D^{\mathrm{sT}}x\right)\right)\right),$$

with $D^{s}$ the dose‐influence matrix of scenario $s\in \left\{0,\ldots ,S\right\}$. This is equivalent to the tractable form

(5) $$\begin{array}{cc} & \max_{x\geq 0}\tau \\ & s\text{.t}.\;\tau \leq \text{log}\left(E_{\left(\alpha ,\rho \right)\in U}\left(TCP\left(\alpha ,\rho ,D^{\mathrm{sT}}x\right)\right)\right)\;\forall s\;\in \;\left\{0,\ldots ,S\right\} \end{array}$$

To account for these geometrical uncertainties, we chose to maximize the worst‐case geometrical scenario rather than optimizing the expectation over the geometrical scenarios, since a worst‐case optimization best resembles the rationale of a PTV margin and corresponds to the way geometrical uncertainties are dealt with in robust optimization in clinical practice, for example for proton therapy planning.

The magnitude of the positional shifts was 2.795 × Σ, which envelops 95% of the scenarios.^31^ Here, Σ is the standard deviation of the systematic errors. The random and systematic errors were set to σ = 5.5 mm and Σ = 3 mm in all directions, which roughly corresponds to data presented by Wolthaus *et al*.^35^ No rotational errors were explicitly considered, although if relevant for a particular tumor type, they could be easily included by adding scenarios.

The optimization of Eq. (5) was implemented in Erasmus‐iCycle, the in‐house developed treatment planning system at Erasmus Medical Center that has been in routine clinical use in combination with Monaco (Elekta, Stockholm) for fully automated treatment planning for head‐and‐neck, lung, cervix, and prostate intensity‐modulated radiation therapy (IMRT) and volumetric‐modulated arc therapy (VMAT) treatments.^25^, ^36^, ^37^ In this study all Erasmus‐iCycle treatment plans consisted of 23 equi‐angular IMRT beams as to mimic VMAT dose distributions.

### 2.B Experimental conditions

For this study 12 non‐small cell lung cancer (NSCLC) patients with planning FDG‐PETCT scans were selected retrospectively. These patients were selected to represent a wide range in tumor locations, volumes, and FDG uptake patterns. In correspondence with the PET boost trial, patients with >50% encasement of the large vessels by the primary tumor were not included, to avoid the risk of large vessel invasion and fatal bleedings.^38^

The FDG distributions were converted to 3D maps ***α*** and ***ρ*** (for details see section 2.B.3). The conversion required three parameters: the TD50 and TD80 of the tumor, defined as the dose required to achieve a TCP of 50% and 80%, and the theoretical gain (Gain_Theo_). Gain_Theo_ was defined as the increase in TCP of a hypothetical DPBN dose distribution that could deliver any dose to any voxel, compared to a uniform dose distribution with the same mean dose.^2^, ^3^ Different values for TD50, TD80, and Gain_Theo_ were considered in this study to investigate how the factors (i) through (v) affect the gains of DPBN compared to NSDE.

Section 2.B.1 describes the choice of the different Gain_Theo_ values; section 2.B.2 describes how TD50 and TD80 were varied to investigate the effect of factor (i) on DPBN, the overall radiosensitivity of the tumor. Section 2.B.3 describes the experiments for factor (ii), different 3D radiosensitivity maps ***α*** and ***ρ***. In section 2.B.4 uncertainty was added to the derived ***α*** and ***ρ*** distributions (factor (iii)). Section 2.B.5 describes factor (iv), the effect of geometrical uncertainties. And finally the effect of temporal changes in radiosensitivity, factor (v), is presented in section 2.B.6.

#### 2.B.1 The theoretical gain

The Gain_Theo_ is likely to represent the upper limit of the gain that could be achieved in practice with DPBN and has been reported in theoretical studies.^2^, ^3^ Considering that one cannot deliver any dose to any voxel, a Gain_Theo_ lower than 0.1 would by definition yield small DPBN gains in real‐world planning situations. This might be realistic, but it would make a study into the dependencies of DPBN impossible. A Gain_Theo_ of 0.3, on the other hand, would imply that by redistribution only, that is, without increasing the integral tumor dose, the TCP could be increased for instance from 0.5 to 0.8, which is likely an overestimation of the true effect. So assuming that DPBN could result in a gain in TCP, the real Gain_Theo_ is likely to lie between 0.1 and 0.3. Since the effect of factors (i) to (v) on the theoretical gain may depend on the theoretical gain itself, different values for Gain_Theo_ were considered: 0.1, 0.2, and 0.3.

#### 2.B.2 Factor (i): The overall radiosensitivity of the tumor

**Fig. 1:**
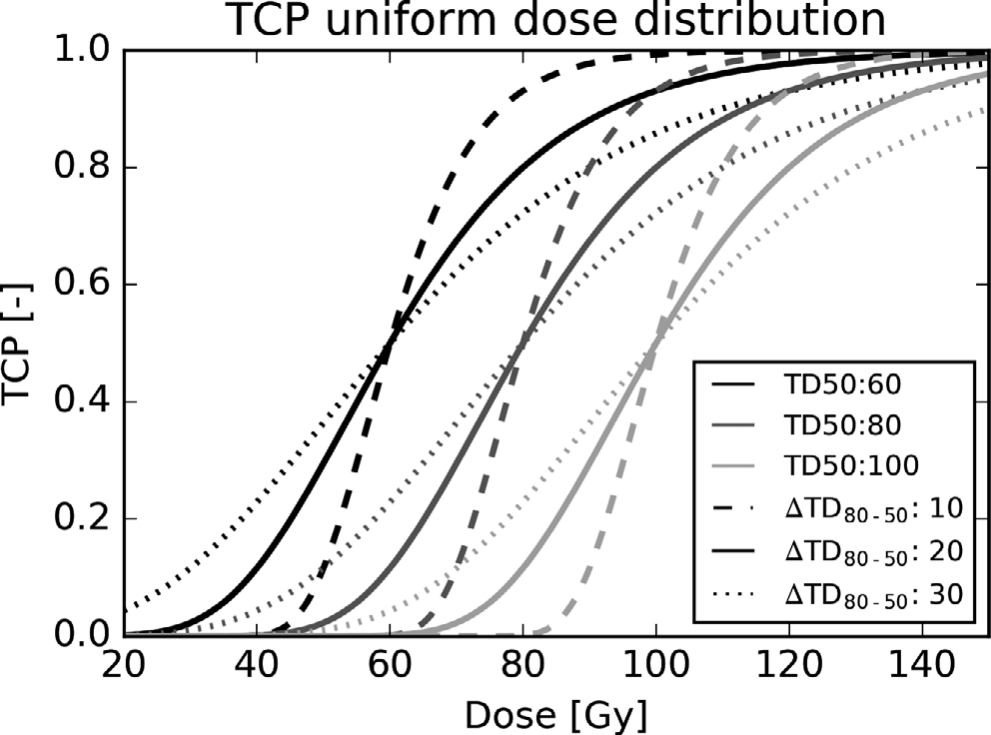
TCP curves for uniform dose distributions for TD50s of 60, 80, and 100 Gy shown in different gray scales. For each TD50 value, three TD80 values were considered of 10, 20, or 30 Gy higher than the TD50, leading to nine different TCP curves for the entire tumor in total.

The radiosensitivity of the entire tumor can be described by TD50 and TD80. Based on reported TD50 values of 72 Gy and 90 Gy for progression‐free survival at 2 yrs for NSCLC,^39^, ^40^ we set the median TD50 to 80 Gy and, to account for variations among patients the range added a TD50 of 60 Gy and 100 Gy, which is roughly 10 Gy lower and higher compared to the reported 72 and 90 Gy.^39^, ^40^ The difference between TD80 and TD50 (ΔTD_80‐50_) was chosen to be 10, 20, or 30 Gy representing steep, moderate, and shallow dose–response relations, see Fig. 1. We chose a wide range of values for TD50 and ΔTD_80‐50_ to ensure that a large fraction of the population was represented within our analysis.

#### 2.B.3 *Factor (ii): The 3D radiosensitivity maps* α *and* ρ

For each combination of TD50, TD80, and Gain_Theo_, the 3D FDG uptake distribution within the clinical target volume (CTV) of each patient (in standardized uptake value, SUV) was converted to 3D radiosensitivity maps ***α*** and ***ρ***, following the approach described in Appendix 1, leading to in total 3^3^ = 27 different ***α*** and ***ρ*** maps per patient.

#### 2.B.4 Factor (iii): Uncertainties in 3D radiosensitivity maps

To determine the effect of uncertainties in radiosensitivity on DPBN, the following procedure was used. Since the 3D radiosensitivity maps ***α*** and ***ρ*** were derived based on TD50, TD80, and Gain_Theo_, uncertainty in ***α*** and ***ρ*** could be described by uncertainty in TD50, TD80, and Gain_Theo_. For this purpose, TD50, ΔTD_80‐50_, and Gain_Theo_ were considered to be random variables that followed Gaussian distributions with means µ_TD50_, µ_TD80‐50_, and µ_GainTheo_, respectively, and standard deviations σ_TD50_, σ_TD80‐50_, and σ_GainTheo_. The means of the distributions were set equal to the median of the parameter ranges as defined above, that is, µ_TD50_ = 80 Gy, µ_TD80‐50_ = 20 Gy, and a µ_Gain_ = 0.2. To investigate the effect of different levels of uncertainty on ΔTCP, σ_TD50_, σ_TD80‐50_, and σ_GainTheo_ were varied from σ_TD50_ = σ_TD80‐50_ = σ_GainTheo_ = 0 (no uncertainty), to a situation with moderate uncertainty (σ_GainTheo_ = 0.05 and σ_TD50_ = σ_TD80‐50_ = 5) and a situation with large uncertainty (σ_GainTheo_ = 0.1 and σ_TD50_ = σ_TD80‐50_ = 10 Gy). For comparison, Martel *et al* found σ_TD50_ between 2.5 Gy and 8 Gy.^39^

The Gaussian distributions of TD_50_, ΔTD_80‐50_, and Gain_Theo_ were discretized in 10 steps varying from −2.5σ to 2.5σ. For each of the experiments, all possible combinations of the 10 values per distribution were considered, leading to 10^3^ = 1000 scenarios per experiment, that is, U in eq. (2) consisted of 1000 scenarios. For each of the 1000 scenarios, the 3D radiosensitivity maps ***α*** and ***ρ*** were calculated according to Appendix 1, to compose the discretized probability density function *p*(***α***,***ρ***) of Eq. (3). To avoid computational problems, TD80 was set at least 5 Gy higher than TD50 and Gain_Theo_ to at least 0.01.

#### 2.B.5 Factor (iv): Geometrical uncertainties

For all experiments performed so far, geometrical uncertainties were accounted for by incorporating them directly into the treatment plan optimization as described in Section 2.A.2. To study the effect of geometrical uncertainties on the gains of DPBN, additional experiments were performed that assumed no geometrical uncertainties (Σ = σ = 0 mm). For these experiments, the ***α*** and ***ρ*** were determined based on the median values of TD50 (80 Gy), ΔTD_80‐50_ (20 Gy), and Gain_Theo_ (0.2), and uncertainty in radiosensitivity was ignored.

#### 2.B.6 Factor (v): Temporal changes

Aerts *et al* showed for a group of 23 NSCLC patients that the location of high and low FDG uptake areas remained stable during treatment^41^ while the maximum SUV did change.^42^ Based on these observations, temporal changes were modeled here by increasing/decreasing the SUV in each voxel by a fixed percentage that was assumed to be representative for the change during treatment. The percentage was varied from −25% to 25%, which corresponded to mean increase of 25% previously observed.^42^ The effect of temporal changes was then evaluated for the DPBN plans that were optimized (assuming no temporal changes) using the median values of TD50 (80 Gy), ΔTD_80‐50_ (20 Gy), and Gain_Theo_ (0.2).

### 2.C Treatment planning

#### 2.C.1 DPBN

Treatment planning consisted for DPBN of optimizing the expected TCP as defined in eq. (5), subject to the constraints of the PET boost trial^4^: maximum dose to the spinal cord <53 Gy; mean lung dose <20 Gy; maximum dose to the brachial plexus <66 Gy, esophagus V_35 Gy_ <80%, maximum dose to the planning organ at risk volume (PRV) around the mediastinal structures (large vessels, heart, trachea, and proximal bronchial tree with 5 mm margin) <94 Gy; maximum dose to the tumor <130 Gy. To ensure a conformal dose distribution and avoid high‐dose spikes, the maximum dose at 1 cm from the CTV was constrained to 60 Gy. To avoid computational problems with extremely low TCP values, the minimum dose to the CTV was constrained to 40 Gy for the experiments that ignored uncertainty in radiosensitivity and slightly higher (50 Gy) for the experiments that acknowledged uncertainty. The distinction in minimal dose was made since the latter encounters uncertainty scenarios where the TCP curve is shifted more toward high doses leading to some scenarios with extremely low TCP values also for a minimal CTV dose of 40 Gy, that would have resulted in numerical problems.

#### 2.C.2 The reference dose distributions

DPBN was compared to two types of dose distributions: (a) a perfectly homogenous dose distribution of 60 Gy, as a surrogate for conventional clinical dose distributions, and (b) a nonselective dose escalation (NSDE) plan. The NSDE dose distributions were obtained using the same optimization strategy as for DPBN, that is, by solving problem (5) with the same OAR constraints, but assuming a fixed $\alpha_{i}$ and $\rho_{i}$ for each tumor voxel. In other words the NSDE dose distributions were obtained by performing a TCP optimization with homogeneous ***α*** and ***ρ*** across the tumor, that is, without requiring FDG distributions or any other patient‐specific biological information.

In case of a homogeneous ***α*** and ***ρ*** across the tumor, ***α*** and ***ρ*** are uniquely defined based only on TD50 and TD80, see Appendix 2. For all NSDE optimizations, ***α*** and ***ρ*** were determined using the median values of TD50 (80 Gy) and ΔTD_80‐50_ (20 Gy). This led to an $\alpha_{i}$of 0.0567 Gy^−1^ for all voxels, patients, and simulations, and a $\rho_{i}$ that depended solely on the number of voxels of the tumor. The NSDE plan was optimized twice, once with and once without acknowledging geometrical uncertainties.

### 2.D Overview of the treatment plans

The various DPBN and NSDE plans that were used to investigate factors (i) to (v) are summarized in Table I. Thirty DPBN plans and two NSDE plans were optimized for each patient, leading to 384 plans in total. TCP represents the worst‐case TCP among the geometrical uncertainty scenarios, that is, τ in problem (5), and Δ TCP represents the difference in TCP between DPBN and NSDE.

**Table I:** Overview of the DPBN and NSDE plans that were used to investigate factors (i) through (v). In total, 30 dose‐painting‐by‐number (DPBN) plans and two nonselective dose escalation (NSDE) plans were generated per patient. The * marks the plan that was used to investigate factor (v), temporal changes.

Factors	Number of plans	TD50 [Gy]	Δ TD_80‐50_ [Gy]	Gain_Theo_ [‐]	Uncertainty radiosensitivity (σ_GainTheo_ [‐] and σ_TD_ [Gy])	Geometrical uncertainty
DPBN						
(i), (ii), (v)*	27	60, 80*, 100	10, 20*, 30	0.1, 0.2*, 0.3	N/A	Yes
(iii)	2	80	20	0.2	0.05 and 5 0.1 and 10	Yes
(iv)	1	80	20	0.2	N/A	No
NSDE						
(i),(ii), (iii), (v)	1	80	20	N/A	N/A	Yes
(iv)	1	80	20	N/A	N/A	No

## 3 Results

**Fig. 2:**
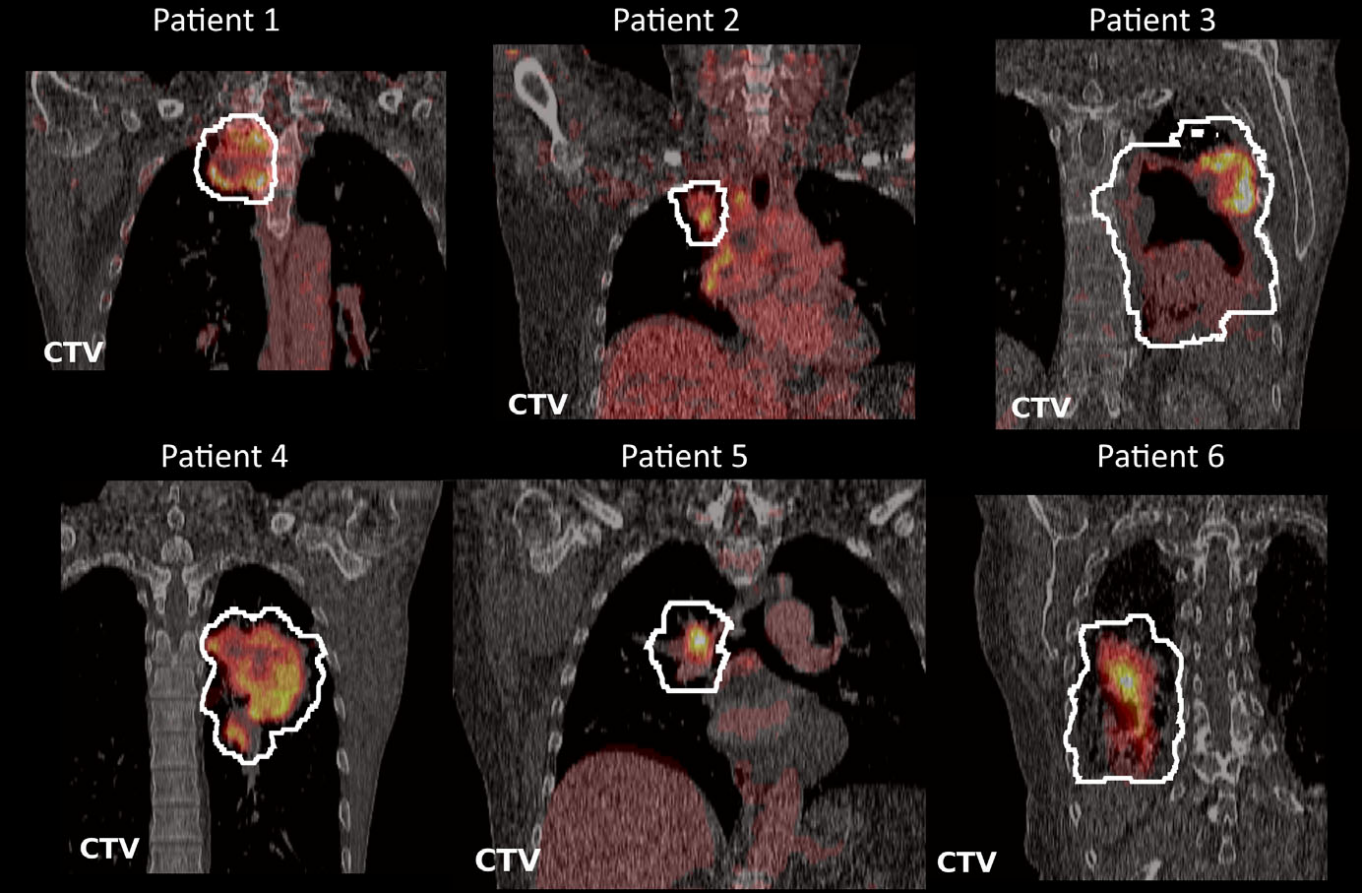
Coronal images of the of the FDG‐PETCT scans of the first six patients through the center of the CTV.

The tumor sizes, locations, and FDG uptake patterns varied considerably among the 12 patients. The median CTV size was 219 cc (range 35–968 cc). The median of the mean and max SUV in the CTV were 2.9 (range 1.7–5.0) and 15.5 (range 7.0–29.7), respectively. The FDG‐PETCT scans of the first six patients are shown in Fig. 2. The scans of all 12 patients can be found in the Supplementary materials S1.

**Fig. 3:**
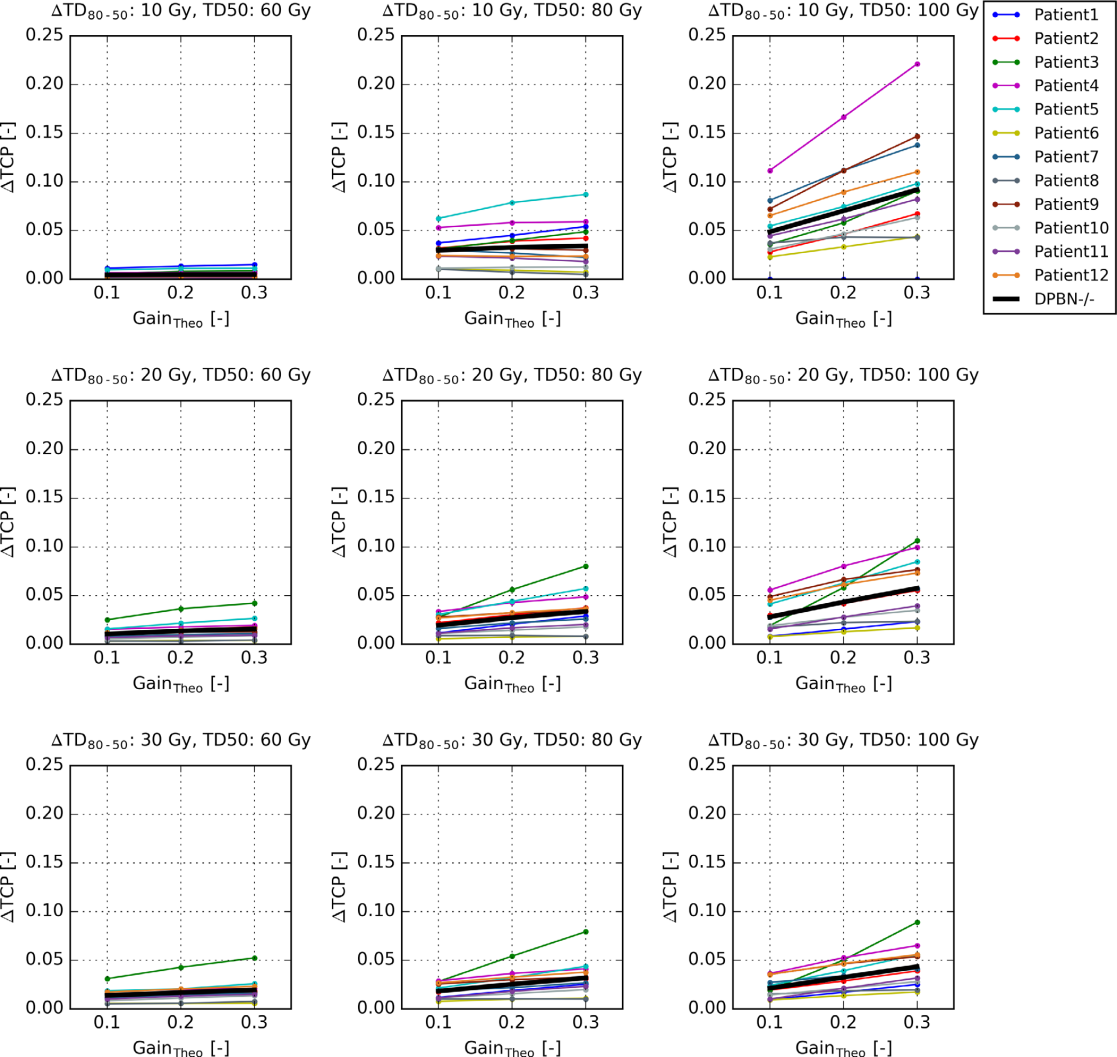
The difference in TCP between the dose‐painting by number (DPBN) plans and the nonselective dose escalation plans (NSDE) for all 12 patients as function of the theoretical gain of DPBN (Gain_Theo_). The rows represent steep, moderate, and shallow TCP relations (expressed by the differences between TD80 and TD50, $\Delta TD_{80‐50}$) and the columns sensitive, moderate, and resistant tumors (expressed by TD50). The thick black lines represent the averages over the 12 patients.

The TCP of DPBN, averaged over all TD50, TD80, and Gain_Theo_ values and patients, was 0.74 ± 0.24, which was 0.54 ± 0.20 (range 0–0.97) higher compared to the TCP of the 60 Gy reference dose distribution of 0.20 ± 0.22. Also NSDE led to large increases in TCP of 0.51 ± 0.20 (range 0–0.96) compared to the reference dose distribution. Compared to NSDE, the gain of DPBN was however moderate: only 0.03 ± 0.03 (range 0–0.22; 95^th^ percentile 0.08). Figure S2 in the supplementary materials shows the TCP for DPBN, NSDE, and the 60 Gy reference dose distribution for all patients, TD50, TD80, and Gain_Theo_ values. The corresponding Δ TCP values are shown in Fig. 3. From here onwards the TCP of DPBN is compared only to the TCP of NSDE.

### 3.A The effect of radiosensitivity of the entire tumor

As expected the radiosensitivity of the entire tumor, reflected by TD50 and TD80, influenced the gain in TCP of DPBN. The TCP gain increased with increasing TD50 from 0.01 ± 0.01 (TD50 = 60) to 0.03 ± 0.02 (TD50 = 80 Gy) to 0.05 ± 0.04 (TD50 = 100 Gy). The gain was also moderately dependent on the steepness of the TCP curves, reflected by ΔTD_80‐50_. Averaged over all patients, TD50 and Gain_Theo_ values, the TCP gain of DPBN decreased with decreasing steepness: From 0.04 ± 0.04 (ΔTD_80‐50_ = 10 Gy) to 0.03 ± 0.02 (ΔTD_80‐50_ = 20 Gy) to 0.02 ± 0.02 (ΔTD_80‐50_ = 30 Gy).

### 3.B The effect of 3D radiosensitivity maps

The combined effect of the 3D radiosensitivity maps and the patient anatomy is expressed by the differences of ΔTCP between the 12 patients. Averaged over all TD50 and TD80 values, the gain in TCP varied between 0.02 ± 0.01 for the patient for whom DPBN was the least favorable (Patient 1) and 0.05 ± 0.05 for the patient for whom DPBN was the most favorable (Patient 4).

### 3.C Uncertainties in 3D radiosensitivity maps

**Fig. 4:**
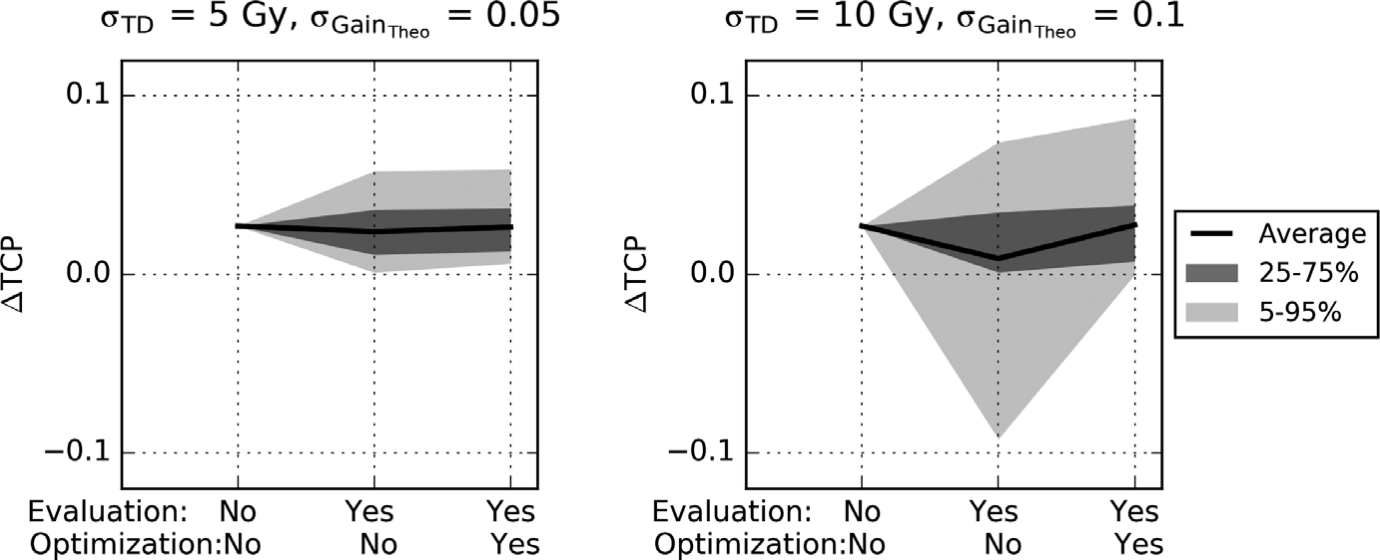
∆TCP for all patients for moderate (left) and considerable (right) uncertainty in radiosensitivity. The x‐axis indicates whether uncertainty was accounted for to calculate the TCP (evaluation) and whether it was accounted for in the optimization. The solid lines represent the mean expected TCPs over all 12 patients. The shaded areas represent 50% and 90% of the cases.

**Fig. 5:**
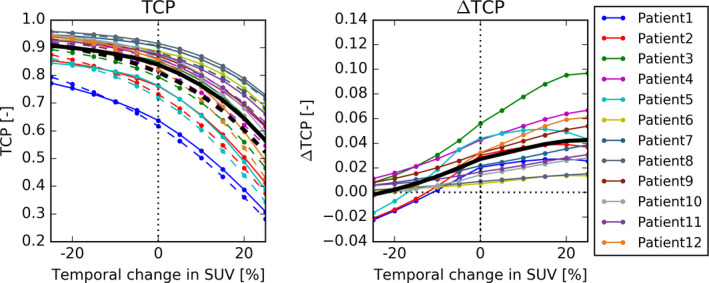
TCP (left) and ∆TCP (right) as function of the temporal change in SUV. The solid lines represent the mean expected TCPs over all 12 patients. A 20% reduction in SUV or more flips the average gain of DPBN from positive to negative. Increasing SUV values led to a slight increase in ∆TCP.

The effect of moderate uncertainty in radiosensitivity (σ_GainTheo_ = 0.05; σ_TD50_ = σ_TD80‐50_ = 5 Gy) on ∆TCP was modest as shown in Fig. 5 (left panel). However, for considerable uncertainty in radiosensitivity (σ_GainTheo_ = 0.10; σ_TD50_ = σ_TD80‐50_ = 10 Gy) (Fig. 4, right panel), the mean expected ∆TCP over all patients reduced from 0.03 to 0.01 when uncertainty was not accounted for in the optimization. For 25% of the experiments, the TCP of DPBN was even lower than with NSDE, and for 5% of the cases the loss in TCP compared to NSDE was more than 0.09. However when uncertainty was acknowledged in the optimization, the mean expected ∆TCP recovered to 0.03 and in only 5% of the cases DPBN led to a lower TCP than NSDE.

### 3.D Geometrical uncertainties

As expected, in the absence of geometrical uncertainties the TCP obtained with DPBN, averaged over all patients, was higher than for NSDE, by 11% on average (0.92 ± 0.02 vs. 0.84 ± 0.08) compared to a situation with geometrical uncertainties. However, the Δ TCP between DPBN and NSDE slightly decreased (0.03 ± 0.01 vs. 0.01 ± 0.01).

### 3.E Temporal changes

Figure 5 shows how the TCP and ∆TCP of the NSDE and DPBN plans change as function of the temporal change in SUV. A 25% increase in SUV led to a decrease in TCP and an increase in ∆TCP of up to 0.01 on average. For a decrease in SUV of 20% or more, the ∆TCP became negative.

### 3.F Dose distributions

The mean dose to the CTV in the nominal scenario of the geometrically robust plans, averaged over all patients, TD50, TD80, and Gain_Theo_, was considerably higher for DPBN (104 Gy) compared to the 60 Gy reference dose distribution, but lower than for NSDE (107 Gy).

**Fig. 6:**
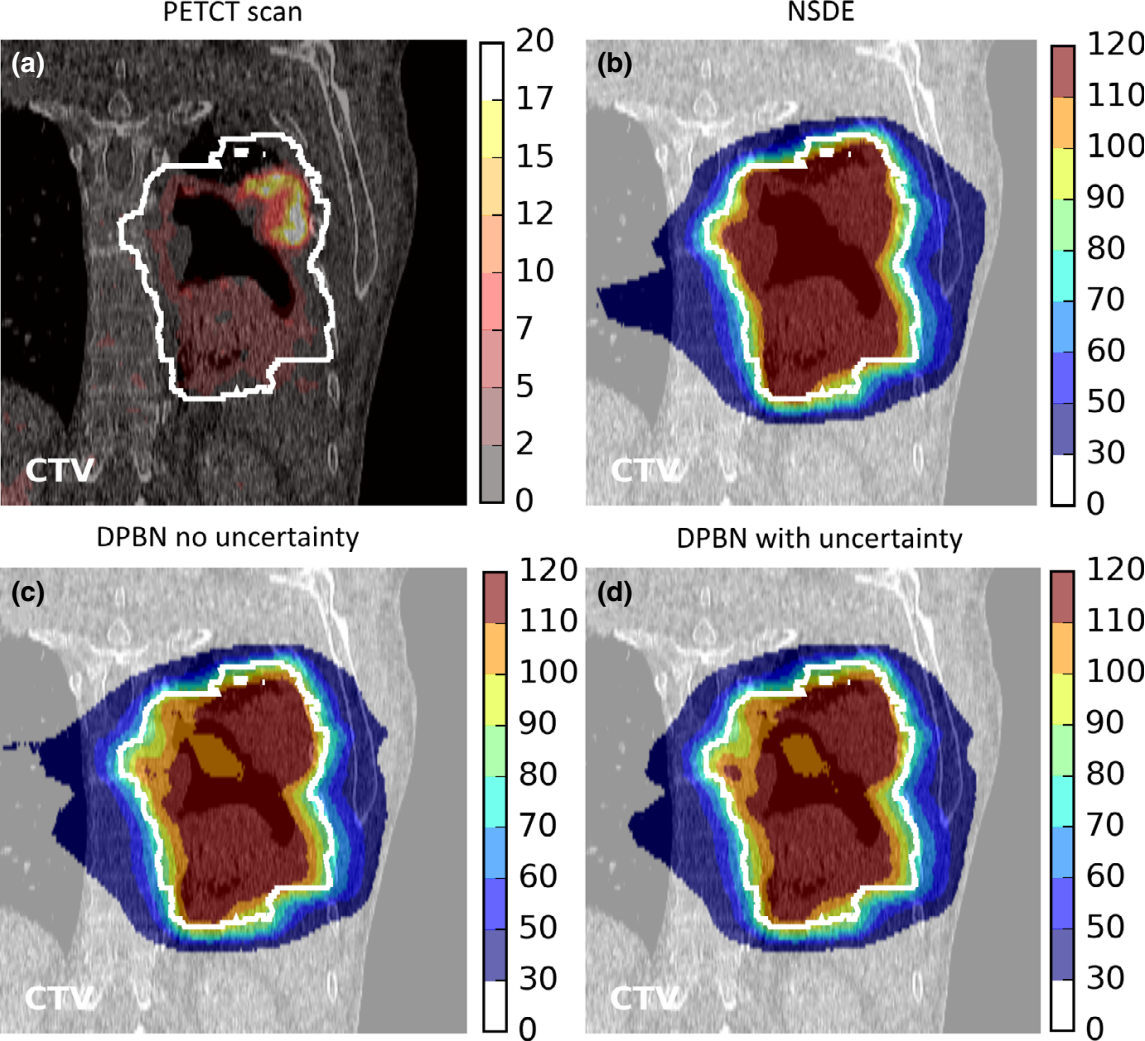
For Patient 3 are shown, on a slice through the center of the CTV, a) the FDG‐PET scan, b) the dose distribution obtained with nonselective dose escalation, c) the dose distribution obtained with dose‐painting‐by‐numbers (DPBN) without acknowledging uncertainty, and d) the dose distribution obtained with DPBN with acknowledging uncertainty (σ_GainTheo_ = 0.10; σ_TD50_ = σ_TD80‐50_ = 10 Gy). All DPBN plans are based on a TD50 of 80 Gy, a TD80 of 100 Gy, and a theoretical DPBN gain in TCP of 0.2 (Gain_Theo_ = 0.2). All plans are geometrically robust. The legends represent SUV (a) or dose in Gy (b, c, d).

Figure 6 shows the FDG‐PET‐CT scan, the NSDE dose distribution, and two DPBN dose distributions without and with acknowledging uncertainty in radiosensitivity for Patient 3. All three plans were geometrically robust. The NSDE plan leads to a more homogeneous dose to the CTV than DPBN. Neither of the two DPBN dose distributions accurately followed the spatial difference in FDG. Excess dose is deposited in regions of the CTV where it is not strictly required, but which is apparently necessary to deposit sufficient dose in other regions. Visually, differences between DPBN dose distributions that did and that did not acknowledge uncertainty in radiosensitivity were small, but apparently effective to compensate for uncertainty (as shown in the right panel of Fig. 5).

## 4 DISCUSSION

In this study, we developed a fully automated robust DPBN optimization strategy that can deal with both positional uncertainties and uncertainties in 3D radiosensitivity maps. It was used to investigate how the theoretical gains of DPBN were affected by variations in patient anatomy, the 3D radiosensitivity maps, uncertainties in radiosensitivity, and patient positioning and temporal changes. For this purpose, a large variation in patient anatomies and the aforementioned parameters was considered. The fact that treatment planning was fully automated allowed us to make an unbiased comparison between DPBN and NSDE. NSDE was implemented as a TCP optimization with fixed and uniform α and ρ across tumor, that is, that did not require voxel or patient‐specific biological information. In total data of 360 DPBN treatment plans and 24 NSDE plans are presented in this study.

We found that a theoretical gain of DPBN of on average 0.2 (range 0.1–0.3) reduced to on average 0.03 (range 0.0–0.22) compared to NSDE when variations in patient anatomy and 3D radiosensitivity distributions were accounted for. This low gain of 0.03 may be in line with presented early results of the PET Boost trial (NCT01024829) that did not demonstrate a considerable difference in overall survival between an isotoxic homogenous dose escalation and FDG‐directed dose escalation.^43^ However, for seven of the 324 cases (2%), we found DPBN did lead to substantial gains in TCP of more than 0.1 compared to NSDE, which suggests that for a narrow bandwidth of patient anatomies and 3D radiosensitivity maps, DPBN could be very effective, also compared to NSDE. To be able to select those patients who could benefit from DPBN, research into deriving intra‐tumor dose–response relationships remains of high interest.^44^

Compared to a uniform reference dose distribution of 60 Gy, on the other hand, both DPBN and NSDE led to huge gains in TCP of on average 0.54 (range 0.0–0.97) and 0.51 (range 0–0.96), respectively. These high gains were a result of the low TCP values of the reference dose distributions (on average 0.2) and the patient anatomies and dose constraints that allowed considerably higher doses to the CTV (104 and 107 Gy on average for DPBN and NSDE respectively) compared to the 60 Gy of the reference dose distribution.

It is challenging to assess which combination of TD50, TD80, and Gain_Theo_ values is most realistic for a given patient. Therefore, we considered a large range of TD50, TD80, and Gain_Theo_ values to yield both sensitive and resistant tumors, with shallow and steep TCP curves and with little or a lot of intra‐tumor variation in radiosensitivity (low and high Gain_Theo_, respectively). Note that the average TCP over all patients and parameter combinations of the 60 Gy reference dose distribution of 0.2 is in line with both the Martel model^39^ and the observed the 2‐year progression‐free survival of advanced stage NSCLC patients.^45^

As an upper bound of the gains of DPBN vs NSDE, one could consider for each patient the best case scenario out of the 27. In that case the mean ΔTCP would be 0.1 ± 0.05 (range 0.04–0.22) over the 12 patients, which is half of the average theoretical gain of 0.2. For 11 of the 12 patients, the highest gain was achieved for very radio‐resistant tumors with steep TCP curves with TD50 of 100 Gy and ΔTD_80‐50_ of 10 Gy. This is understandable since (a) the TD50 marks the steepest part of the TCP curve, where little more (effective use of) dose can have a large effect on TCP, (b) the TD50 of 100 Gy was close to the average mean dose of NSDE and DPBN, and (c) a ΔTD80‐50 of 10 was the highest steepness considered in this study.

To assess the effect of various degrees of uncertainty in radiosensitivity (factor (c)), we varied the probability distribution on α and ρ. The distribution was governed by assumed Gaussian distributions of TD50, Δ TD_80‐50_, and Gain_Theo_, for which the standard deviations were varied. Uncertainty in radiosensitivity could lead to a considerable loss in Δ TCP. However, the loss could be recovered almost completely by accounting for the uncertainty directly in the optimization even for the distributions with a large standard deviation.

Our results showed that in the presence of geometrical uncertainties (factor (iv)), the gains of DPBN were higher than in the absence of geometrical uncertainties. This may seem counterintuitive, as geometrical uncertainties impose additional restrictions on dose delivery and were therefore expected to limit the potential gains of DPBN. However, the additional restrictions led to a decrease in TCP for both DPBN and NSDE toward a steeper part of the TCP curve, where a small increase in (effective) dose could have a substantial effect on TCP. Consequently, the TCP difference between DPBN and NSDE increased when geometrical uncertainties were acknowledged.

Temporal changes (factor (v)) influenced the gains of DPBN. If the SUV decreased during treatment, the TCP decreased toward the steeper part of the curve leading to an increase in ∆TCP of up 0.01 on average. On the other hand, when the SUV distribution decreased during treatment, ∆TCP rapidly decreased to 0.

The DPBN dose distributions shown in Fig. 6 did not show a strong visual correlation between the SUV and the delivered dose. Apparently depositing a high dose in regions with a low SUV can be beneficial in terms of TCP maximization. This suggests that DPBN strategies that determine a desired dose per voxel a priori based on 3D radiosensitivity maps and then minimize the difference between the desired and achievable dose would yield considerably different dose distributions than DPBN strategies that directly optimize the TCP.

The 324 different 3D radiosensitivity maps ***α*** and ***ρ*** were created based on FDG‐PET scans of 12 NSCLC patients with the only assumption a linear relation between $\alpha_{i}$ and SUV and a constant $\rho_{i}$ within the tumor. For an individual case, ***α*** and ***ρ*** were governed then by the choice of TD50, Δ TD_80‐50_, and Gain_Theo_. More complicated relations between SUV and $\alpha_{i}$ and $\rho_{i}$ than linear would have required estimating additional parameters to describe such a relationship, which in turn would have required introducing more parameters than only TD50, Δ TD_80‐50_, and Gain_Theo_. This implies including more unknowns in an already uncertain relationship and was therefore deemed undesirable. In case SUV would have been converted linearly to $\rho_{i}$ instead of to $\alpha_{i}$, the desired dose difference would have been steered by the log of the difference in SUV. Since the log of the SUV difference is less extreme than the SUV difference itself, we expect this to lead to a less extreme dose redistribution and therefore smaller differences between DPBN and NSDE. Potentially in reality there may be no clear relation between FDG‐PET scans and 3D intra‐tumor radiosensitivity maps. In that case the used 3D radiosensitivity maps obtained here should be considered merely as examples of what radiosensitivity distributions across the tumor could look like.

The current study was restricted to the primary tumor, while potentially affected lymph nodes were ignored. Including the lymph nodes would mean that the achievable tumor dose would be lower than the average mean dose of 102 Gy for DPBN found in this study. Since the largest gains of DPBN are expected when the achievable dose is at the steep part of the TCP curve, this would imply that the largest gains would not be achieved for a TD50 of 100 Gy, as suggested by this study, but more likely for a lower TD50. A similar effect is expected when dose constraints would have been stricter than those that were used here. Since additional restrictions of tumor size and more stringent dose constraints would apply to both NSDE and DPBN, it is expected that the effect on the TCP gains between DPBN and NSDE reported here would be small. Although compared to the 60 Gy reference dose distribution, the gains of DPBN would be smaller.

Several factors that may influence the benefits of DPBN were outside the scope of this study. First, we assumed that the VCP was independent between voxels, which is likely an over simplification. Second, the current robust TCP‐based DPBN implementation is a fluence‐based optimization only. The choice of VMAT segments could influence the achievable dose distributions, but the effects are expected to be moderate and could become in principle arbitrarily small.^46^ In general, any additional complexity that is not accounted in the DPBN optimization would likely reduce the gains of DPBN compared to those presented here. For NSDE, $\alpha_{i}$ was fixed among all patients, voxels, and simulations, and $\rho_{i}$ depended solely on tumor size. $\alpha_{i}$ and $\rho_{i}$ were chosen based on the median values of TD50 and TD80. It could be of interest for future studies to determine if different fixed choices of $\alpha_{i}$ and $\rho_{i}$ for NSDE could have increased the TCP of NSDE further and therefore lowered the gains of DPBN.

## 5 Conclusion

Based on a large range of patient anatomies and 3D radiosensitivity maps, our results suggest that the gains of DPBN can be considerable compared to a 60 Gy reference dose distribution, but small (0.03 in TCP) compared to NSDE for most patients. In only in 2% of our experiments, gains in TCP > 0.1 compared to NSDE were achieved. The gains varied depending on the radiosensitivity of the entire tumor and the distribution of radiosensitivity across the tumor in combination with patient anatomy. Uncertainty in radiosensitivity reduced the gains considerably, but could be effectively compensated for by accounting for uncertainty directly in the optimization. Using the robust DPBN system developed here, the optimal DPBN dose distribution and estimates of the gains of DPBN can be derived for any patient when their 3D intra‐tumor radiosensitivity maps are known. This allows for the selection of patients that might benefit from DPBN. Nonselective dose escalation could be an effective strategy to increase TCP without requiring biological information of the tumor.

## Conflict of Interest

The authors have no conflict to disclose.

## Supporting information

**Fig S1.** Axial, Coronal, and sagittal FDG‐PET‐CT images through the center of the CTV for all 12 patients.
**Fig S2.** TCP values of the dose‐painting by number plans (DPBN), nonselective dose escalation plans (NSDE), and the 60 Gy reference dose distributions for all 12 patients as function of the theoretical gain of DPBN. The different rows represent steep, moderate, and shallow TCP relations (expressed by the differences between TD80 and TD50, Δ***TD***80 − 50) and the columns, sensitive, moderate, and resistant tumors (expressed by TD50). The thick black lines represent the averages over the 12 patients. For the most radio‐resistant tumors (TD50 = 100 Gy), the TCP of the reference dose distribution was around 0, though still substantial for NSDE and DPBN.

## Appendix 1 Deriving αi and ρ based on TD50, ΔTD_80‐50_, and Gain_Theo_ for DPBN

As shown below, TD50, TD80, and Gain_Theo_ constitute three equations. Three equations allows a transformation from the SUV of each voxel, $s_{i}$, to the radiosensitivity parameters, $\alpha_{i}$ and $\rho_{i}$, that is characterized by a maximum of three unknowns. We chose a linear relation between $s_{i}$ and $\alpha_{i}$, that is, $\alpha_{i}=c_{1}s_{i}+c_{0}$, requiring two of the three unknowns ($c_{0}$ and $c_{1}$). ρ is assumed to be independent of SUV, such that ρ itself was the third unknown. The impact of choosing $\alpha_{i}$ to be linearly related to SUV and $\rho_{i}$ to be constant across the tumor is discussed in the Discussion section.

Based on TD50 and TD80, it follows that for a uniform dose distribution with $d_{i}=TD50$.

(A1) $$0.5=\exp\left(‐\sum_{i}\rho_{i}\;\exp\left(‐\alpha_{i}TD50\right)\right),$$

and for $d_{i}=TD80$

(A2) $$0.8=\exp\left(‐\sum_{i}\rho_{i}\;\exp\left(‐\alpha_{i}TD80\right)\right).$$

Based on the TCP of DPBN in an ideal scenario with a mean tumor dose equal to the TD50 of the tumor, the Gain_Theo_ can be written as

(A3) $$0.5+Gain_{\mathrm{Theo}}=\exp\left(‐\sum_{i}\rho_{i}\;\exp\left(‐\alpha_{i}d_{i}\right)\right),$$

and since the mean dose is equal to TD50 it follows that

(A4) $$TD50=\frac{1}{N}\left(d_{1}+\sum_{i=2...N}d_{i}\right)$$

By inserting $\alpha_{i}=c_{1}s_{i}+c_{0}$ and $\rho_{i}=\rho$, Eq. (A2) becomes.

$$0.8=\exp\left(‐\rho \sum_{i}\exp\left(‐\left(c_{1}s_{i}TD80+c_{0}TD80\right)\right)\right)$$

$$0.8=\exp\left(‐\rho \sum_{i}\exp\left(‐c_{1}s_{i}TD80\right)\exp\left(‐c_{0}TD80\right)\right)$$

$$0.8=\exp\left(‐\rho \;\exp\left(‐c_{0}TD80\right)\sum_{i}\exp(‐\left(c_{1}s_{i}TD80\right)\right),$$

which yields and expression for *ρ*:

(A5) $$\rho =\frac{‐\ln\;0.8}{\exp\left(‐c_{0}TD80\right)\sum_{i}\exp(‐\left(c_{1}s_{i}TD80\right)}.$$

A similar expression can be derived based on Eq. (A1):

(A6) $$\rho =\frac{‐\ln\;0.5}{\exp\left(‐c_{0}TD50\right)\sum_{i}\exp(‐\left(c_{1}s_{i}TD50\right)}$$

By combining (A5) and (A6), it follows that $c_{0}$ can be expressed as a function of $c_{1}$:

$$\frac{‐\ln\;0.5}{\exp\left(‐c_{0}TD50\right)\sum_{i}\exp(‐\left(c_{1}s_{i}TD50\right)}=\frac{‐\ln\;0.8}{\exp\left(‐c_{0}TD80\right)\sum_{i}\exp(‐\left(c_{1}s_{i}TD80\right)}$$

$$\frac{\ln\;0.5}{\ln\;0.8}=\frac{\exp\left(‐c_{0}TD50\right)\sum_{i}\exp(‐\left(c_{1}s_{i}TD50\right)}{\exp\left(‐c_{0}TD80\right)\sum_{i}\exp(‐\left(c_{1}s_{i}TD80\right)}$$

$$\frac{\ln\;0.5}{\ln\;0.8}=\frac{\exp{\left(‐c_{0}\right)}^{TD50}\sum_{i}\exp(‐\left(c_{1}s_{i}TD50\right)}{\exp{\left(‐c_{0}\right)}^{TD80}\sum_{i}\exp(‐\left(c_{1}s_{i}TD80\right)}$$

$$\frac{\ln\;0.5}{\ln\;0.8}=\frac{\exp{\left(‐c_{0}\right)}^{TD50‐TD80}\sum_{i}\exp(‐\left(c_{1}s_{i}TD50\right)}{\sum_{i}\exp(‐\left(c_{1}s_{i}TD80\right)}$$

$$\exp{\left(‐c_{0}\right)}^{TD50‐TD80}=\frac{\sum_{i}\exp(‐\left(c_{1}s_{i}TD80\right)}{\sum_{i}\exp(‐\left(c_{1}s_{i}TD50\right)}\frac{\ln\;0.5}{\ln\;0.8}$$

(A7)
$$c_{0}=‐\ln\left({\left(\frac{\sum_{i}\exp(‐\left(c_{1}s_{i}TD80\right)}{\sum_{i}\exp(‐\left(c_{1}s_{i}TD50\right)}\frac{\ln\;0.5}{\ln\;0.8}\right)}^{\frac{1}{TD50‐TD80}}\right).$$

For a tumor with a nonuniform distribution of radiation sensitivity, the optimal dose distribution for a given mean dose is achieved if the probability of surviving cells is the same for each voxel.^47^ Therefore, eq. (A3) can be rewritten as

$$‐\rho \;\exp\left(‐\left(c_{1}s_{i}+c_{0}\right)d_{i}\right)=‐\rho \;\exp\left(‐\left(c_{1}s_{1}+c_{0}\right)d_{1}\right)=‐\rho \;\exp\left(‐\left(c_{1}s_{2}+c_{0}\right)d_{2}\right)\ldots etc$$

Since ρ is assumed to be constant across the tumor, eq. (A3) simplifies to

$$0.5+Gain_{\mathrm{Theo}}=\exp\left(‐\rho N\exp(‐\left(c_{1}s_{1}+c_{0}\right)d_{1})\right).$$

It follows that the dose in a voxel $d_{i}$ can be expressed as a function of the dose in the first tumor voxel $d_{1}$.

$$d_{i}=\frac{c_{1}s_{1}+c_{0}}{c_{1}s_{i}+c_{0}}d_{1}.$$

Inserting this into eq. (A4) yields

$$TD50=\frac{1}{N}\left(d_{1}+\sum_{i=2..N}d_{i}\right)=\frac{1}{N}\left(d_{1}+d_{1}\sum_{i=2..N}\frac{c_{1}s_{1}+c_{0}}{c_{1}s_{i}+c_{0}}\right).$$

It follows that

(A8) $$d_{1}=\frac{TD50N}{1+\sum_{i=2..N}\frac{c_{1}s_{1}+c_{0}}{c_{1}s_{i}+c_{0}}}.$$

By inserting (A5), (A7), and (A8), eq (A3) becomes:

(A9) $$0.5+Gain_{\mathrm{Theo}}=\exp\left(‐\rho N\exp(‐\left(c_{1}s_{1}+c_{0}\right)d_{1})\right).$$

At this point an expression is achieved with $c_{1}$ as the only unknown. This means that for given values of Gain_Theo_, TD50, and TD80, the parameters that link FDG uptake to $\alpha_{i},$ that is, $c_{1}$ and $c_{0}$ can be determined.

Equation (A9) was solved numerically for the TD50, ΔTD_80‐50_, and the Gain_Theo_ values of interest, with the additional constraints that $c_{1}s_{i}+c_{0}\;>\;0$ for all i, to ensure only non‐negative $\alpha_{i}$; with $c_{1}\;<\;0$ such that high SUV voxels are more resistant (lower $\alpha_{i}$) than low SUV voxels; and $d_{i}\;>\;0$ for all i. Note that once $c_{1}$ is known, $c_{0}$ and ρ can be determined using Eqs. (A7) and (A5) and $\alpha_{i}$ can be determined for each voxel. To increase computational efficiency, the SUV distribution within the tumor was discretized using 250 bins from the minimal SUV to the 99^th^ percentile. Equation (A7) in combination with the additional constraints does not by definition lead to a single solution for $c_{1}$, although for all parameter combinations in this study it did.

## Appendix 2 Deriving αi and ρ based on TD50, ΔTD_80‐50_, and Gain_Theo_ for NSDE

For a tumor with a uniform α and ρ, α and ρ can be determined solely based on the TD50 and TD80 as follows from the following equations

$$\ln\left(0.5\right)=‐N\rho \;\exp\left(‐\alpha TD_{50}\right)$$

$$\ln\left(0.8\right)=‐N\rho \;\exp\left(‐\alpha TD_{80}\right),$$

which can be rewritten as

$$\ln\left(0.5\right)=\ln\left(0.8\right)\exp\left(\left(TD_{80}‐TD_{50}\right)\alpha \right),$$

thus

$$\alpha =\frac{\text{ln(}\ln\;(0.5)/\ln\;(0.8))}{TD_{80}‐TD_{50}}$$

and

$$\rho =‐\frac{\ln\;\left(0.8\right)}{\text{Nexp}\left(‐\alpha TD_{80}\right)}.$$

## References

[1] Bentzen SM, Gregoire V. Molecular imaging‐based dose painting: a novel paradigm for radiation therapy prescription. Semin Radiat Oncol. 2011;21:101–110. 21356478 10.1016/j.semradonc.2010.10.001 PMC3052283

[2] Sovik A, Malinen E, Bruland OS, Bentzen SM, Olsen DR. Optimization of tumour control probability in hypoxic tumours by radiation dose redistribution: a modelling study. Phys Med Biol. 2007;52:499–513. 17202629 10.1088/0031-9155/52/2/013

[3] F Petit S, Dekker ALAJ, Seigneuric R, et al. Intra‐voxel heterogeneity influences the dose prescription for dose‐painting with radiotherapy: a modelling study. Phys Med Biol. 2009;54:2179–2196. 19293465 10.1088/0031-9155/54/7/022

[4] Laprie A, Ken S, Filleron T, et al. Dose‐painting multicenter phase III trial in newly diagnosed glioblastoma: the SPECTRO‐GLIO trial comparing arm A standard radiochemotherapy to arm B radiochemotherapy with simultaneous integrated boost guided by MR spectroscopic imaging. BMC Cancer. 2019;19:167. 30791889 10.1186/s12885-019-5317-x PMC6385401

[5] Aerts HJWL, van Baardwijk AAW, Petit SF, et al. Identification of residual metabolic‐active areas within individual NSCLC tumours using a pre‐radiotherapy 18Fluorodeoxyglucose‐PET‐CT scan. Radiother Oncol. 2009;91:386–392. 19329207 10.1016/j.radonc.2009.03.006 PMC4693609

[6] Borren A, Groenendaal G, Moman MR, et al. Accurate prostate tumour detection with multiparametric magnetic resonance imaging: Dependence on histological properties. Acta Oncol (Stockholm, Sweden). 2014;53:88–95. 10.3109/0284186X.2013.837581 24041257

[7] Chang JH, Wada M, Anderson NJ, et al. Hypoxia‐targeted radiotherapy dose painting for head and neck cancer using (18)F‐FMISO PET: A biological modeling study. Acta Oncol (Stockholm, Sweden). 2013;52:1723–1729. 10.3109/0284186X.2012.759273 23317145

[8] Grosu A‐L, Souvatzoglou M, Röper B, et al. Hypoxia imaging with FAZA‐PET and theoretical considerations with regard to dose painting for individualization of radiotherapy in patients with head and neck cancer. Int J Radiat Oncol Biol Phys. 2007;69:541–551. 17869667 10.1016/j.ijrobp.2007.05.079

[9] Laprie A, Catalaa I, Cassol E, et al. Proton magnetic resonance spectroscopic imaging in newly diagnosed glioblastoma: predictive value for the site of postradiotherapy relapse in a prospective longitudinal study. Int J Radiat Oncol Biol Phys. 2008;70:773–781. 18262090 10.1016/j.ijrobp.2007.10.039

[10] Vanderstraeten B, Duthoy W, De Gersem W, De Neve W, Thierens H. [18F]fluoro‐deoxy‐glucose positron emission tomography ([18F]FDG‐PET) voxel intensity‐based intensity‐modulated radiation therapy (IMRT) for head and neck cancer. Radiother Oncol. 2006;79:249–258. 16564588 10.1016/j.radonc.2006.03.003

[11] Yaromina A, Granzier M, Biemans R, et al. A novel concept for tumour targeting with radiation: Inverse dose‐painting or targeting the “Low Drug Uptake Volume”. Radiother Oncol. 2017;124:513–520. 28502472 10.1016/j.radonc.2017.04.020

[12] Zamboglou C, Thomann B, Koubar K, et al. Focal dose escalation for prostate cancer using 68Ga‐HBED‐CC PSMA PET/CT and MRI: a planning study based on histology reference. Radiat Oncol. 2018;13:81. 29716617 10.1186/s13014-018-1036-8 PMC5930745

[13] Gronlund E, Johansson S, Nyholm T, Thellenberg C, Ahnesjo A. Dose painting of prostate cancer based on Gleason score correlations with apparent diffusion coefficients. Acta Oncol (Stockholm Sweden). 2018;57:574–581. 10.1080/0284186X.2017.1415457 29260950

[14] Bradshaw T, Fu R, Bowen S, Zhu J, Forrest L, Jeraj R. Predicting location of recurrence using FDG, FLT, and Cu‐ATSM PET in canine sinonasal tumors treated with radiotherapy. Phys Med Biol. 2015;60:5211–5224. 26083082 10.1088/0031-9155/60/13/5211 PMC6415760

[15] van Elmpt W, De Ruysscher D, van der Salm A, et al. The PET‐boost randomised phase II dose‐escalation trial in non‐small cell lung cancer. Radiother Oncol. 2012;104:67–71. 22483675 10.1016/j.radonc.2012.03.005

[16] Bowen SR, Flynn RT, Bentzen SM, Jeraj R. On the sensitivity of IMRT dose optimization to the mathematical form of a biological imaging‐based prescription function. Phys Med Biol. 2009;54:1483–1501. 19218733 10.1088/0031-9155/54/6/007 PMC2858011

[17] Sterpin E, Differding S, Janssens G, Geets X, Gregoire V, Lee JA. Generation of prescriptions robust against geometric uncertainties in dose painting by numbers. Acta Oncol (Stockholm, Sweden). 2015;54:253–260. 10.3109/0284186X.2014.930171 24991892

[18] Das SK, Miften MM, Zhou S, et al. Feasibility of optimizing the dose distribution in lung tumors using fluorine‐18‐fluorodeoxyglucose positron emission tomography and single photon emission computed tomography guided dose prescriptions. Med Phys. 2004;31:1452–1461. 15259648 10.1118/1.1750991

[19] Thorwarth D, Eschmann SM, Paulsen F, Alber M. Hypoxia dose painting by numbers: A planning study. Int J Radiat Oncol Biol Phys. 2007;68:291–300. 17448882 10.1016/j.ijrobp.2006.11.061

[20] Alber M, Thorwarth D. Multi‐modality functional image guided dose escalation in the presence of uncertainties. Radiother Oncol. 2014;111:354–359. 24880742 10.1016/j.radonc.2014.04.016

[21] Kim Y, Tome WA. Dose‐painting IMRT optimization using biological parameters. Acta Oncol (Stockholm, Sweden). 2010;49:1374–1384. 10.3109/02841861003767539 20429729

[22] Petit SF, Aerts HJWL, van Loon JGM, et al. Metabolic control probability in tumour subvolumes or how to guide tumour dose redistribution in non‐small cell lung cancer (NSCLC): An exploratory clinical study. Radiother Oncol. 2009;91:393–398. 19328570 10.1016/j.radonc.2009.02.020

[23] van Schie MA, Steenbergen P, Dinh CV, et al. Repeatability of dose painting by numbers treatment planning in prostate cancer radiotherapy based on multiparametric magnetic resonance imaging. Phys Med Biol. 2017;62:5575–5588. 28557799 10.1088/1361-6560/aa75b8

[24] Her EJ, Haworth A, Reynolds HM, et al. Voxel‐level biological optimisation of prostate IMRT using patient‐specific tumour location and clonogen density derived from mpMRI. Radiat Oncol. 2020;15:172. 32660504 10.1186/s13014-020-01568-6 PMC7805066

[25] Breedveld S, Storchi PR, Heijmen BJ. The equivalence of multi‐criteria methods for radiotherapy plan optimization. Phys Med Biol. 2009;54:7199–7209. 19920305 10.1088/0031-9155/54/23/011

[26] Alber M, Paulsen F, Eschmann SM, Machulla HJ. On biologically conformal boost dose optimization. Phys Med Biol. 2003;48:N31–N35. 12587912 10.1088/0031-9155/48/2/404

[27] Hoffmann AL, den Hertog D, Siem AY, Kaanders JH, Huizenga H. Convex reformulation of biologically‐based multi‐criteria intensity‐modulated radiation therapy optimization including fractionation effects. Phys Med Biol. 2008;53:6345–6362. 18941280 10.1088/0031-9155/53/22/006

[28] Romeijn HE, Dempsey JF, Li JG. A unifying framework for multi‐criteria fluence map optimization models. Phys Med Biol. 2004;49:1991–2013. 15214537 10.1088/0031-9155/49/10/011

[29] Prékopa A. On logarithmic concave measures and functions. Acta Sci Math. 1973;34:335–343.

[30] Breedveld S, van den Berg B, Heijmen B. An interior‐point implementation developed and tuned for radiation therapy treatment planning. Comput Optim Appl. 2017;68:209–242.

[31] van Herk M, Remeijer P, Rasch C, Lebesque JV. The probability of correct target dosage: dose‐population histograms for deriving treatment margins in radiotherapy. Int J Radiat Oncol Biol Phys. 2000;47:1121–1135. 10863086 10.1016/s0360-3016(00)00518-6

[32] Beckham WA, Keall PJ, Siebers JV. A fluence‐convolution method to calculate radiation therapy dose distributions that incorporate random set‐up error. Phys Med Biol. 2002;47:3465–3473. 12408475 10.1088/0031-9155/47/19/302

[33] Leong J. Implementation of random positioning error in computerised radiation treatment planning systems as a result of fractionation. Phys Med Biol. 1987;32:327–334. 3575415 10.1088/0031-9155/32/3/002

[34] van Herk M, Witte M, van der Geer J, Schneider C, Lebesque JV. Biologic and physical fractionation effects of random geometric errors. Int J Radiat Oncol Biol Phys. 2003;57:1460–1471. 14630286 10.1016/j.ijrobp.2003.08.026

[35] Wolthaus JWH, Sonke J‐J, van Herk M, et al. Comparison of different strategies to use four‐dimensional computed tomography in treatment planning for lung cancer patients. Int J Radiat Oncol Biol Phys. 2008;70:1229–1238. 18313530 10.1016/j.ijrobp.2007.11.042

[36] Della Gala G, Dirkx MLP, Hoekstra N, et al. Fully automated VMAT treatment planning for advanced‐stage NSCLC patients. Strahlenther Onkol. 2017;193:402–409. 28314877 10.1007/s00066-017-1121-1 PMC5405101

[37] Heijmen B, Voet P, Fransen D, et al. Fully automated, multi‐criterial planning for Volumetric Modulated Arc Therapy – An international multi‐center validation for prostate cancer. RadiotherOncol. 2018;128:343–348. 10.1016/j.radonc.2018.06.023 29970259

[38] van Diessen J, De Ruysscher D, Sonke J‐J, et al. The acute and late toxicity results of a randomized phase II dose‐escalation trial in non‐small cell lung cancer (PET‐boost trial). Radiother Oncol. 2019;131:166–173. 30327236 10.1016/j.radonc.2018.09.019

[39] Martel MK, Ten Haken RK, Hazuka MB, et al. Estimation of tumor control probability model parameters from 3‐D dose distributions of non‐small cell lung cancer patients. Lung Cancer. 1999;24:31–37. 10403692 10.1016/s0169-5002(99)00019-7

[40] Kong F‐M, Ten Haken RK, Schipper MJ, et al. High‐dose radiation improved local tumor control and overall survival in patients with inoperable/unresectable non‐small‐cell lung cancer: Long‐term results of a radiation dose escalation study. Int J Radiat Oncol Biol Phys. 2005;63:324–333. 16168827 10.1016/j.ijrobp.2005.02.010

[41] Aerts HJWL, Bosmans G, van Baardwijk AAW, et al. Stability of 18F‐deoxyglucose uptake locations within tumor during radiotherapy for NSCLC: A prospective study. Int J Radiat Oncol Biol Phys. 2008;71:1402–1407. 18234432 10.1016/j.ijrobp.2007.11.049

[42] van Baardwijk A, Bosmans G, Dekker A, et al. Time trends in the maximal uptake of FDG on PET scan during thoracic radiotherapy. A prospective study in locally advanced non‐small cell lung cancer (NSCLC) patients. Radiother Oncol. 2007;82:145–152. 17258339 10.1016/j.radonc.2007.01.007

[43] Lalezari F, Lambrecht M, Lewensohn R, et al. The PET‐boost trial: Isotoxic homogeneous or FDG‐directed dose escalation in stage II‐III NSCLC. Radiother Oncol. 2020;152:S345.

[44] Yan D, Chen S, Krauss DJ, et al. Inter/intra‐tumoral dose response variations assessed using FDG‐PET/CT feedback images: Impact on tumor control and treatment dose prescription. Radiother Oncol. 2020;154:235–242. 33035624 10.1016/j.radonc.2020.09.052

[45] Aupérin A, Le Péchoux C, Rolland E, et al. Meta‐analysis of concomitant versus sequential radiochemotherapy in locally advanced non‐small‐cell lung cancer. J Clin Oncol. 2010;28:2181–2190. 20351327 10.1200/JCO.2009.26.2543

[46] Bortfeld TR, Kahler DL, Waldron TJ, Boyer AL. X‐ray field compensation with multileaf collimators. Int J Radiat Oncol Biol Phys. 1994;28:723–730. 8113118 10.1016/0360-3016(94)90200-3

[47] Brahme A, Agren AK. Optimal dose distribution for eradication of heterogeneous tumours. Acta Oncol (Stockholm, Sweden). 1987;26:377–385. 10.3109/02841868709104364 3426851

