# Supplementary material for: Robust dose‐painting‐by‐numbers vs. nonselective dose escalation for non‐small cell lung cancer patients
Source: Med Phys. 2021 May 14;48(6):3096–108. doi: 10.1002/mp.14840 (PMC8411426; doi:10.1002/mp.14840)

Figure S1

Axial, Coronal, and sagittal FDG-PET-CT images through the center of the CTV for all 12 patients

Patient 1	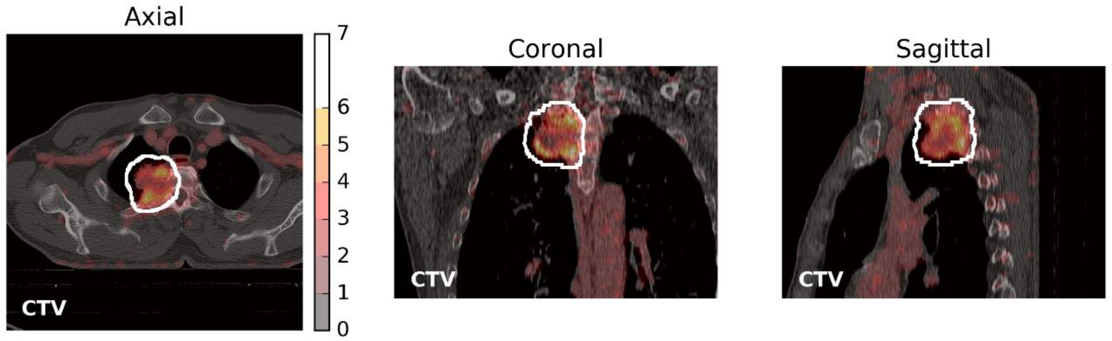
Patient 2	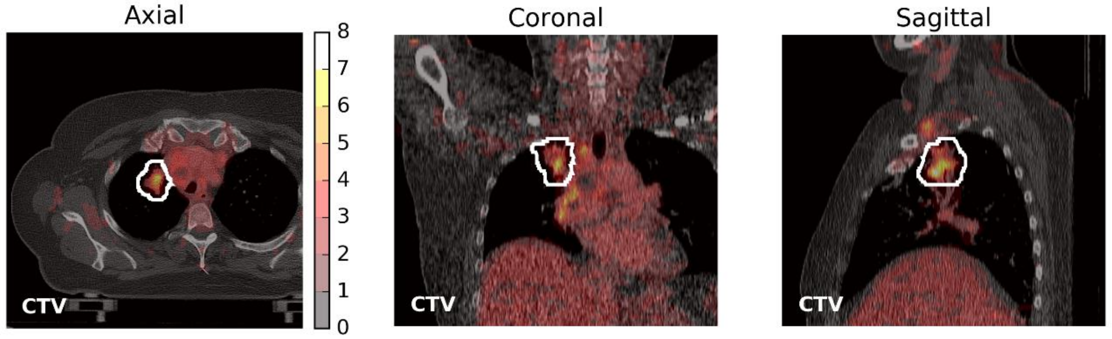
Patient 3	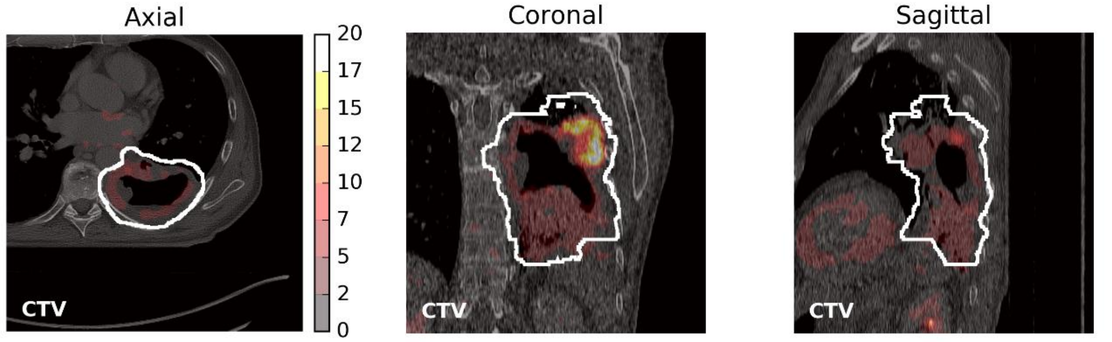
Patient 4	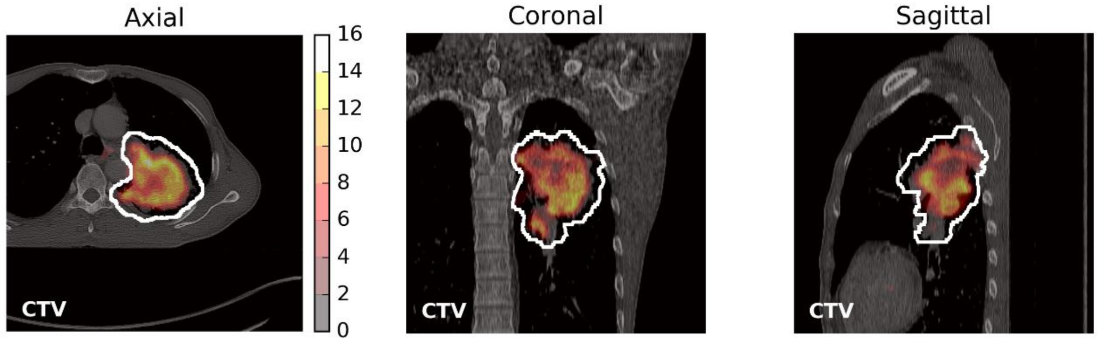

Patient 5	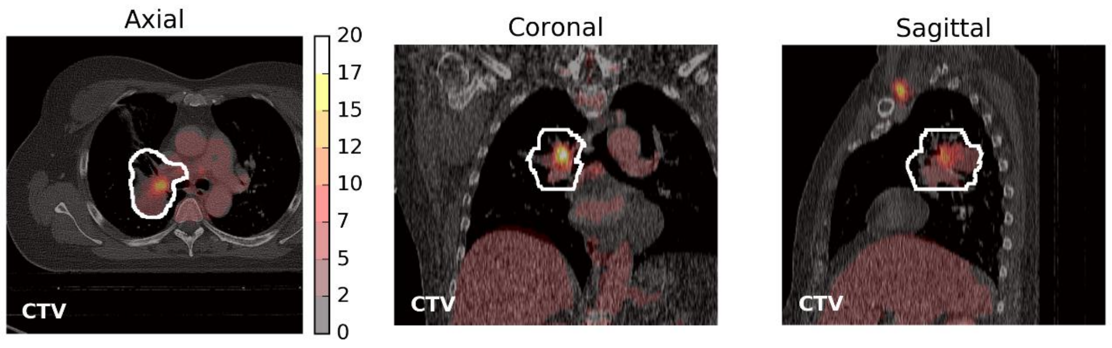
Patient 6	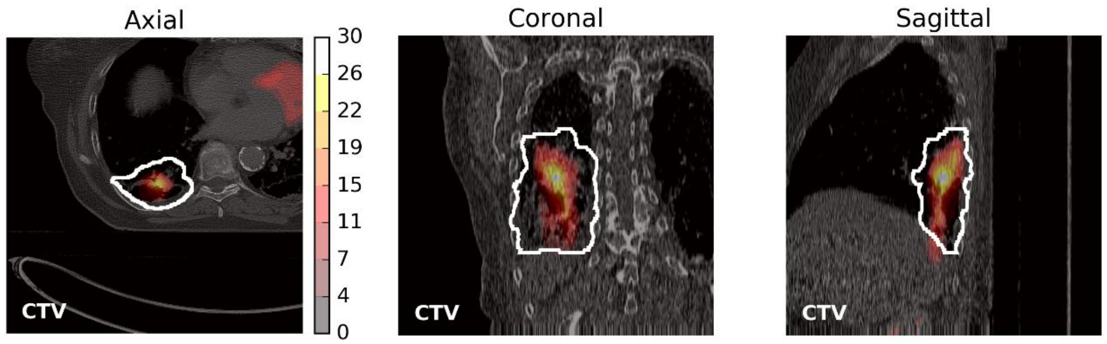
Patient 7	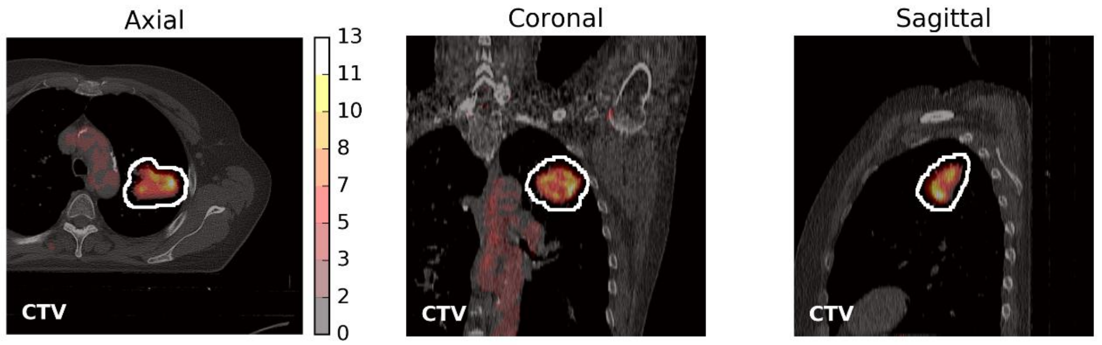
Patient 8	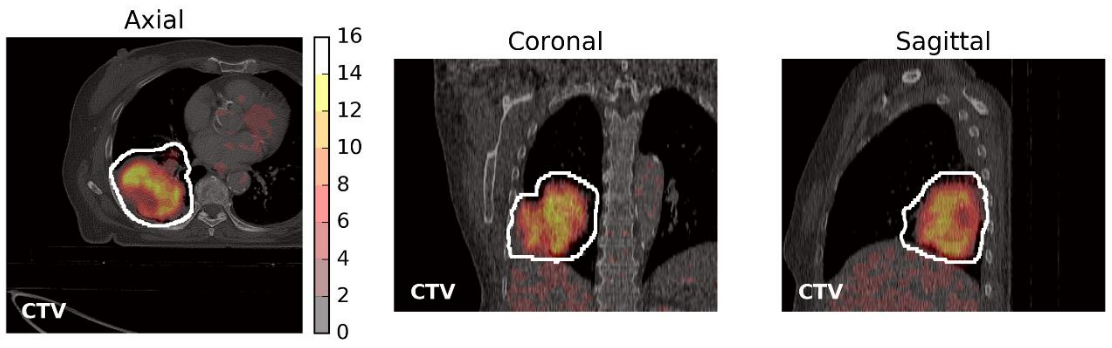

Patient 9	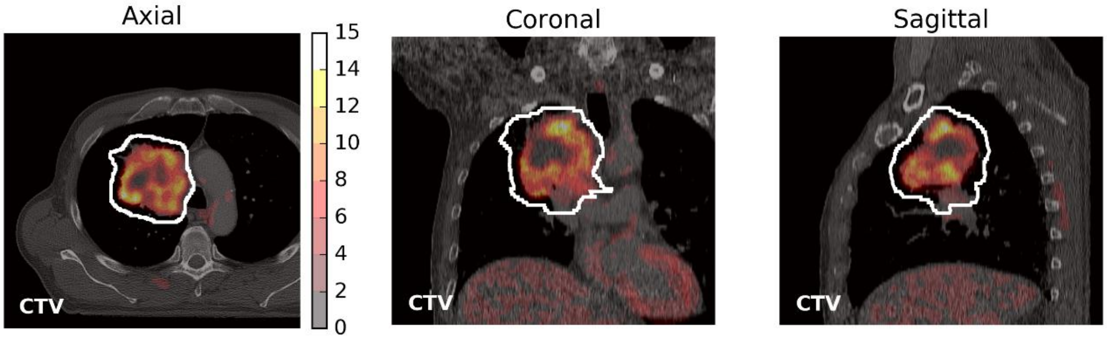
Patient 10	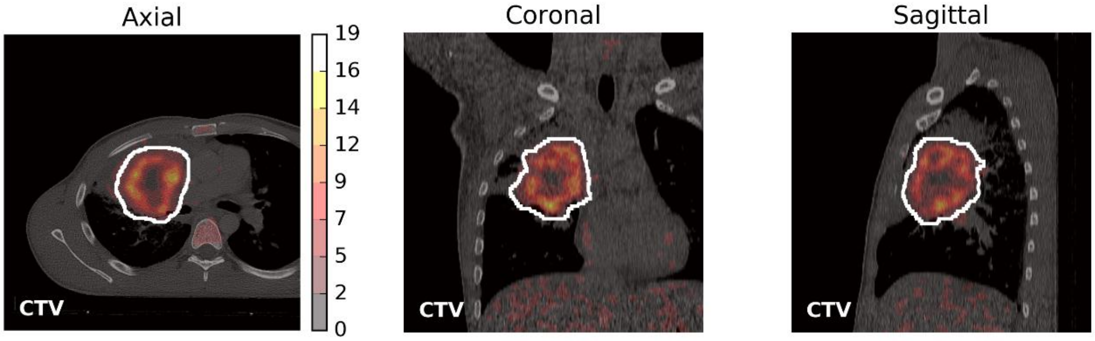
Patient 11	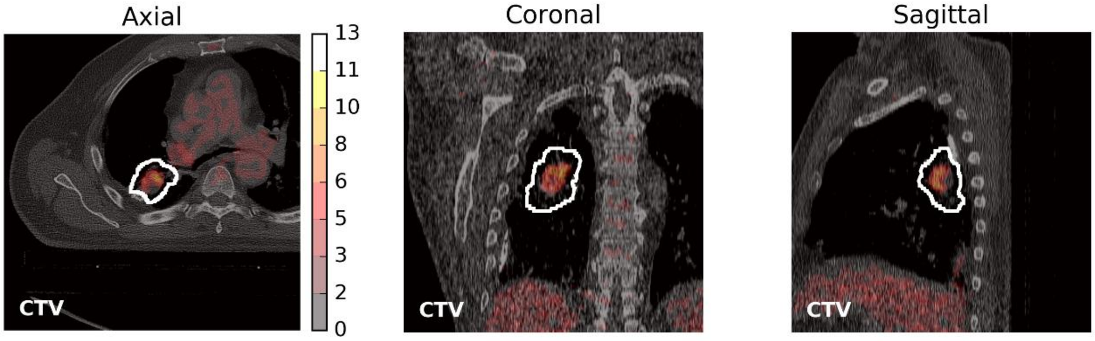
Patient 12	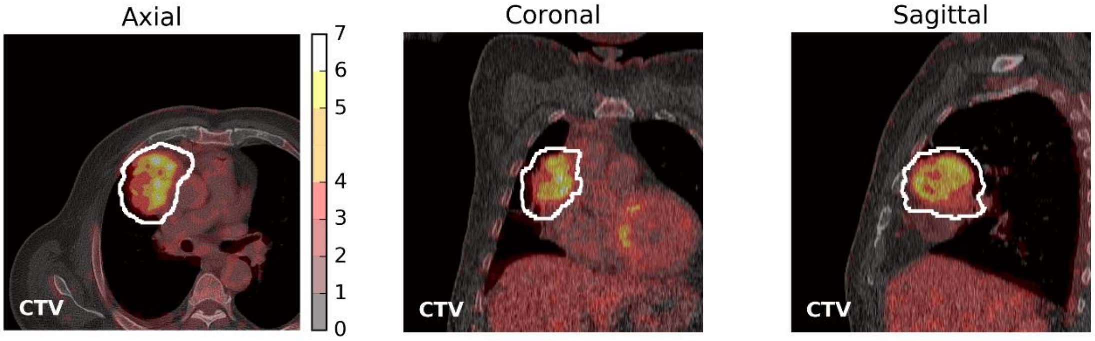

Figure S2

TCP values of the dose-painting by numbers plans (DPBN), non-selective dose escalation plans (NSDE) and the 60 Gy reference dose distributions for all 12 patients as function of the theoretical gain of DPBN. The different rows represent steep, moderate and shallow TCP relations (expressed by the differences between TD80 and TD50, ΔTD_{80-50}) and the columns, sensitive, moderate and resistant tumors (expressed by TD50). The thick black lines represent the averages over the 12 patients. For the most radio-resistant tumors (TD50 = 100 Gy) the TCP of the reference dose distribution was around 0, though still substantial for NSDE and DPBN

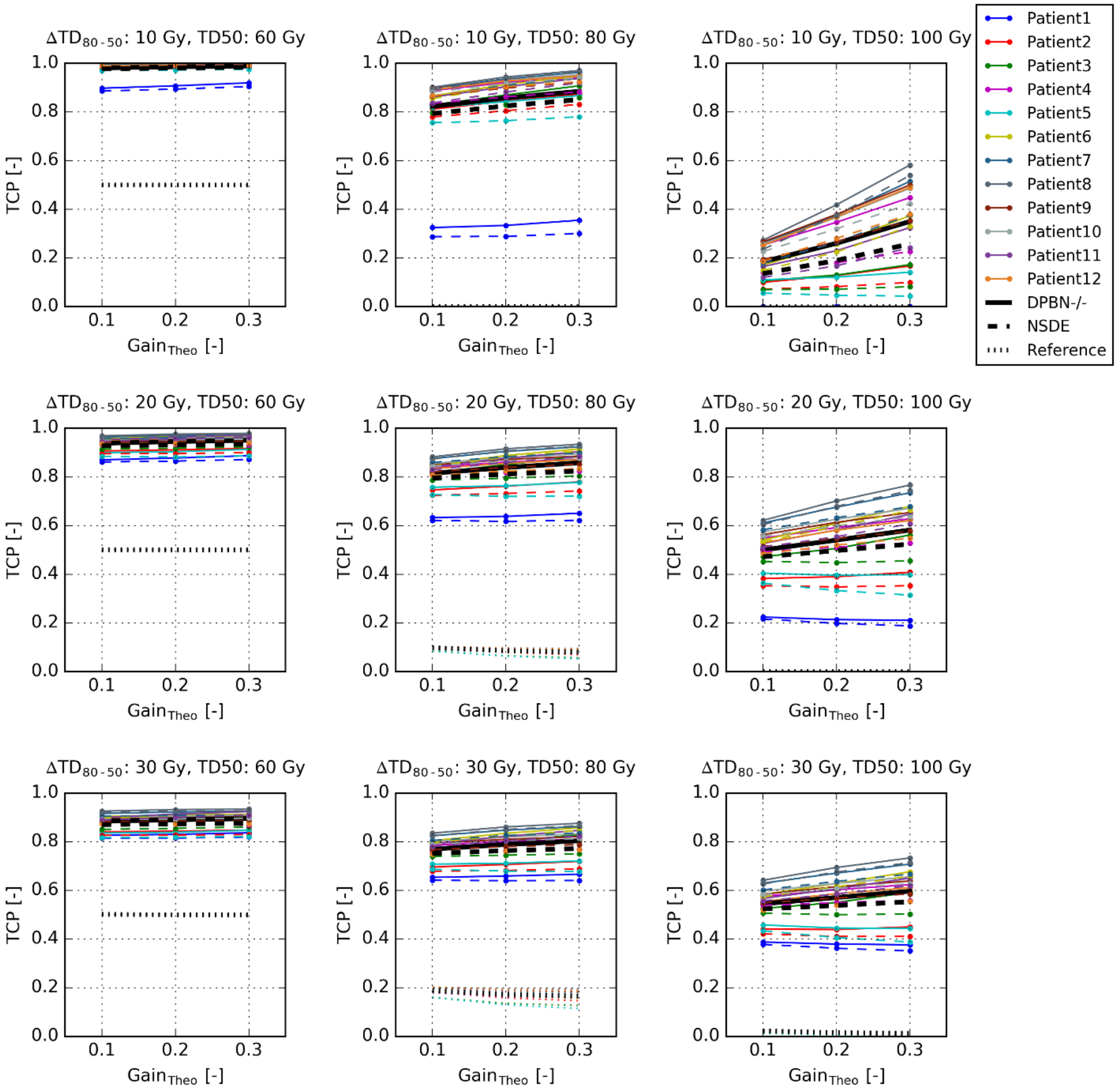

Supplement: Supplementary file 1 — Fig S1. Axial, Coronal, and sagittal FDG‐PET‐CT images through the center of the CTV for all 12 patients. Fig S2. TCP values of the dose‐painting by number plans (DPBN), nonselective dose escalation plans (NSDE), and the 60 Gy reference dose distributions for all 12 patients as function of the theoretical gain of DPBN. The different rows represent steep, moderate, and shallow TCP relations (expressed by the differences between TD80 and TD50, ΔTD80 − 50) and the columns, sensitive, moderate, and resistant tumors (expressed by TD50). The thick black lines represent the averages over the 12 patients. For the most radio‐resistant tumors (TD50 = 100 Gy), the TCP of the reference dose distribution was around 0, though still substantial for NSDE and DPBN. [file MP-48-3096-s001.pdf]
